# Daytime REM sleep affects emotional experience but not decision choices in moral dilemmas

**DOI:** 10.1038/s41598-017-11530-4

**Published:** 2017-09-11

**Authors:** Nicola Cellini, Lorella Lotto, Carolina Pletti, Michela Sarlo

**Affiliations:** 1 Department of General Psychology, University of Padova, Padova, Italy; 2 Department of Developmental Psychology and Socialization, University of Padova, Padova, Italy

## Abstract

Moral decision-making depends on the interaction between automatic emotional responses and rational cognitive control. A natural emotional regulator state seems to be sleep, in particular rapid eye movement (REM) sleep. We tested the impact of daytime sleep, either with or without REM, on moral decision. Sixty participants were presented with 12 sacrificial (6 Footbridge- and 6 Trolley-type) and 8 everyday-type moral dilemmas at 9 AM and at 5 PM. In sacrificial dilemmas, participants had to decide whether or not to kill one person to save more people (utilitarian choice), and to judge how morally acceptable the proposed choice was. In everyday-type dilemmas, participants had to decide whether to endorse moral violations involving dishonest behavior. At 12 PM, 40 participants took a 120-min nap (17 with REM and 23 with NREM only) while 20 participants remained awake. Mixed-model analysis revealed that participants judged the utilitarian choice as less morally acceptable in the afternoon, irrespective of sleep. We also observed a negative association between theta activity during REM and increased self-rated unpleasantness during moral decisions. Nevertheless, moral decision did not change across the day and between groups. These results suggest that although both time and REM sleep may affect the evaluation of a moral situation, these factors did not ultimately impact the individual moral choices.

## Introduction

A moral dilemma involves a conflict in choosing between two undesirable alternatives, both of which have aversive consequences and neither of which clearly emerges as the right choice, such as in choosing between killing one person and letting many people die^1, 2^. Classical examples of this situation are the Trolley and the Footbridge dilemmas. In the Trolley dilemma, the only way to save five workmen from a runaway trolley is to pull a lever redirecting the trolley onto a sidetrack, where it will kill a single workman. In the Footbridge dilemma, the only way to save the five workmen is to push a large man off an overpass onto the track, where he will die while his body will stop the trolley. Despite the same cost/benefit ratio, moral judgments in the two dilemmas appear to be driven by different principles, as most people judge that pulling the lever in the Trolley dilemma is morally acceptable, whereas pushing the man in the Footbridge dilemma is not^3^.

According to the dual-process theory of moral judgment^4, 5^, both automatic emotional responses and rational cognitive control play crucial roles in driving moral decisions. When contemplating moral dilemmas such as the Trolley and the Footbridge problems, people automatically experience an immediate negative emotional reaction to the thought of causing harm to others. If sufficiently strong, this aversive reaction will override the rational cognitive computation of costs/benefits of harming others, resulting in the rejection of utilitarian resolutions (i.e., sacrificing one person to save a larger number of people). If the emotional reaction is weak and/or the cognitive resources are fully recruited, the cognitive processing will dominate decision-making, resulting in the approval of utilitarian outcomes. Footbridge-type dilemmas elicit a strong emotional engagement, which is likely triggered by the involvement of intentional and direct harmful actions towards the victim (e.g., pushing a man off the overpass to stop the trolley). In contrast, in Trolley-type dilemmas, harm is a foreseen side-effect of a goal and is caused in an indirect way (such as when pulling a lever to divert the trolley)^6, 7^. In the latter case, emotion is more weakly engaged, so that cognitive control might emerge in guiding decision-making towards utilitarian resolutions (e.g., approving of killing one person to save more lives). At neural levels, these two competing processing systems are thought to be implemented, respectively, by emotional brain networks, involving mainly the amygdala and the ventromedial prefrontal cortex (vmPFC), and cognitive control networks, including the dorsolateral prefrontal cortex and the inferior parietal lobe^4, 5^.

A consequence of the dual-process theory is that reducing emotional responses should lead to an increase in the cognitive-controlled decisions, which can be operationalized in a higher number of utilitarian responses. Indeed, studies testing individuals with psychopathy, characterized by emotional deficits and propensity to immoral behavior, show vmPFC hypoactivation and are more likely to endorse utilitarian outcomes^8^. Moreover, neuropsychological studies consistently showed an atypically high number of utilitarian responses to Footbridge-type dilemmas in patients with focal lesions to the vmPFC^9, 10^, suggesting a causal role played by emotional brain networks in rejecting utilitarian resolutions. Besides these studies in atypical populations, very few studies involving resolution of moral dilemmas have directly manipulated emotional state in normotypical individuals. For example, Valdesolo and DeSteno^11^, induced positive or neutral affect in their participants using a 5-min comedy clip or a segment of a documentary, before asking them to solve a Footbridge and a Trolley dilemma. They observed higher utilitarian responses in the Footbridge dilemma in participants who watched the funny clip.

An alternative approach to manipulating emotional processing in normotypical individuals is to capitalize spontaneous “emotional regulation” intervention, such as sleeping. Indeed, as proposed by the *Sleep to Remember, Sleep to Forget* (SRSF) model^12^, the suppression of adrenergic activity during rapid eye movement (REM) sleep, coupled with activation in amygdala-vmPFC-hippocampal networks, is implicated in valence re-evaluation and implicit emotion regulation during the integration of negative experiences into long-term memory, possibly mediated by typical theta oscillations (4–8 Hz). In other words, the intensity of emotional experiences at both the neural and subjective levels is decreased during REM sleep while preserving the content of the experience.

Although the SRSF model is a very influential and cited model on sleep and emotions, and offers a good framework to empirically test hypotheses about the role of sleep, in particular REM sleep, on emotional processing, not all findings support the selective role of REM sleep in emotional memory processing, with some studies identifying a synergistic relationship between non-rapid eye movement (NREM) and REM sleep in the processing of emotional information^13^. These findings suggest that during NREM the re-activation of memory traces coupled with reduced amygdala-vmPFC-hippocampal connectivity may allow for consolidation of the information in the cortex independent of emotional tone. In the subsequent REM, the concurrent activation of amygdala-vmPFC-hippocampal circuit, may help synthesize the emotional tone of the newly integrated memories via theta activity^14^. Thus, both NREM and REM may together facilitate emotional regulation.

**Figure 1:**
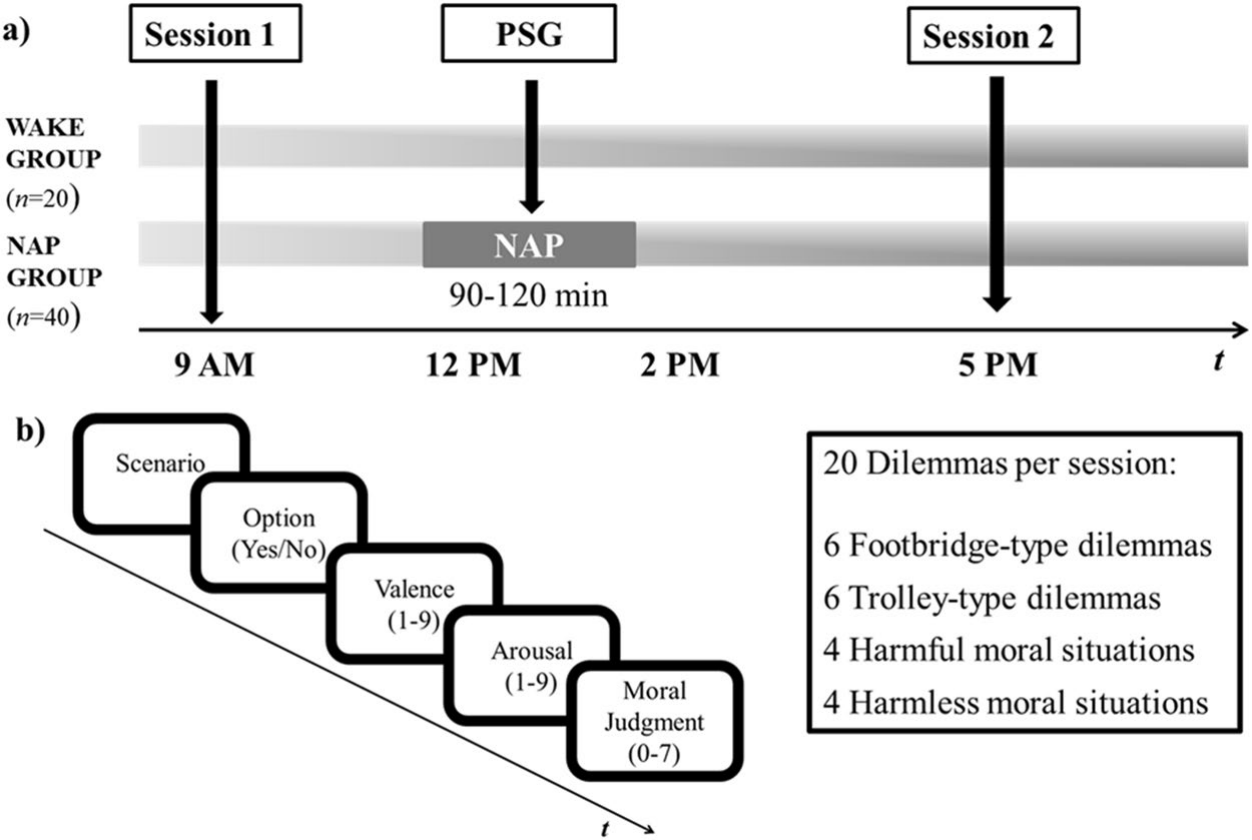
(**a)** Schematic representation of the experimental procedure. All participants were presented with one set of 20 moral problems at 9 AM (Session 1). At 12 PM participants in the NAP group took either a 90- or a 120-min nap. At 5 PM the second set of 20 moral problems were presented to all participants (Session 2). (**b**) In each session, the moral problems were presented in a randomized order, including 6 Footbridge-type and 6 Trolley-type dilemmas, and 4 harmful and 4 harmless everyday situations. Each dilemma was presented as a scenario, followed by a hypothetical action that could be performed (Option). Participants were required to choose whether or not they would perform the proposed action (Yes/No). Afterward, participants had to rate how they felt while they were deciding in terms of unpleasantness/pleasantness (Valence) and calm/activation (Arousal). Lastly, participants had to rate to what extent the proposed action was morally acceptable (not at all/completely).

On these grounds, here we tested the effect of sleep on the decision-making and emotional experience in moral dilemmas, by capitalizing on a nap paradigm^15, 16^, which allowed us to modulate the presence or absence of REM sleep in an ecological way (i.e., without forcing the awakening of participants during their sleep). Specifically, we aimed to exploit the changes in emotional experience and in decision-making on a moral dilemma task after a daytime nap containing REM sleep (REM group) as compared to sleep with no REM (NoREM group, in which both the emotional and the cognitive control networks will be deactivated) and to an equivalent period of time awake (WAKE group; see Fig. 1). In addition, the nap paradigm allows us to test the impact of accumulated sleepiness and fatigue throughout a “typical” working day (9 AM–5 PM) on moral decisions, and to assess whether a nap may modulate these effects.

We hypothesized that:

- In the test task, during re-exposure to comparable moral dilemmas, the REM-group will show increased utilitarian choices and decreased perceived unpleasantness and arousal as compared to baseline and relative to both the NoREM- and the WAKE-group. These effects are expected to be stronger (or even exclusive) for Footbridge- than for Trolley-type dilemmas.
- In order to demonstrate a direct effect of REM sleep physiology, we hypothesize that in the REM-group percentage of time spent in REM and the total power of the EEG theta band during REM sleep might emerge as significant predictors of the behavioral responses and subjective emotional reactivity. Specifically, based on the relevant literature^17^, we hypothesized that time spent in REM and the total power of the theta band will show positive associations with the reduction of emotional reactivity and with the increase in utilitarian responses.

In addition, to test the above hypotheses with more realistic moral situations, we employed a set of everyday moral conflict situations involving no deaths, but describing situations in which the choice is between a personal advantage and either others’ welfare (*harmful situations*, e.g., stealing) or fulfillment of moral obligations (*harmless situations*, e.g., lying).

## Results

### Sleep parameters

Prior to the experimental day, participants were randomly assigned to a NAP (N = 40; F = 21; 22.48 ± 2.24 years) or a WAKE (N = 20, F = 12; 23.25 ± 2.24 years) condition. Participants in the NAP group took either a 90-min or a 120-min polysomnographic-recorded nap at 12 PM (see Table 1). These durations were chosen in order to increase the likelihood of obtaining naps with and without REM sleep since shorter naps tend to have less or no REM sleep than longer naps^18^. Based on post-hoc sleep stage scoring, participants in the NAP group were further divided into either the REM (N = 17; F = 10; 22.41 ± 1.73 years) or NoREM (N = 23; F = 11; 22.52 ± 2.59 years) groups. Of the 18 participants originally assigned to the 90-min NAP group, 5 participants produced REM sleep and 13 showed NREM sleep only, whereas, among the 22 participants assigned to the 120-min NAP group, 12 participants showed REM sleep. Sleep summary is reported in Table 1.

**Table 1:** Means and SDs for PSG measures in the NoREM and REM groups. *T* and *p* values of between groups *t- test* comparisons are reported.

	NoREM *N* = 23	REM *N* = 17	*t*	*p*
TST (min)	57.20 ± 19.72	75.71 ± 16.17	−3.16	0.003
SOL (min)	9.96 ± 6.63	12.32 ± 7.91	−1.03	0.311
WASO (min)	19.13 ± 11.56	8.53 ± 10.14	3.02	0.005
SE (%)	65.40 ± 17.52	78.83 ± 12.28	−2.70	0.010
N1 (min)	15.13 ± 9.21	16.32 ± 8.14	−0.43	0.673
N2 (min)	34.70 ± 16.30	33.94 ± 13.57	0.16	0.877
N3 (min)	7.37 ± 9.80	14.47 ± 16.47	−1.70	0.097
REM (min)	—	10.97 ± 6.52	—	—
N2 Frontal Spindles (#)	75.94 ± 36.84	72.35 ± 34.53	0.31	0.757
N2 Frontal Spindles (density)	2.23 ± 0.41	2.11 ± 0.43	0.89	0.380
N2 Central Spindles (#)	75.33 ± 36.31	70.59 ± 30.27	0.44	0.665
N2 Central Spindles (density)	2.23 ± 0.46	2.11 ± 0.39	0.86	0.393
N3 Frontal SWA (LN μV^2^/Hz)	7.51 ± 0.29	7.63 ± 0.50	−0.76	0.454
REM Frontal Theta (LN μV^2^/Hz)	—	3.83 ± 0.62	—	—

LN = natural logarithm; REM = Rapid Eye Movement; SE = Sleep Efficiency; SOL = Sleep Onset Latency; SWA = Slow Wave Activity; TST = Total Sleep Time; WASO = Wake After Sleep Onset.

### Sleepiness and fatigue

**Figure 2:**
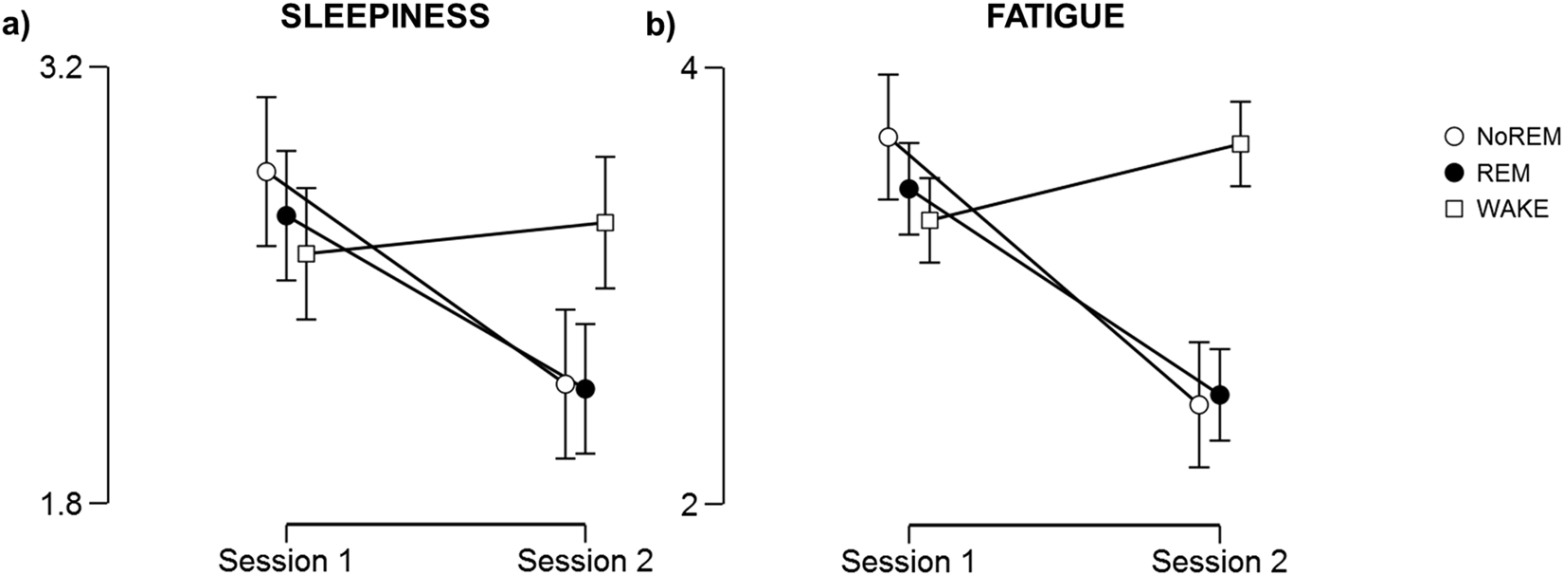
(**a**) Sleepiness and (**b**) fatigue level as a function of group and session. NoREM and REM groups showed a reduction of both sleepiness and fatigue level in the Session 2, after the nap, whereas the WAKE group maintained the same level across the two sessions. Error bars represent standard error.

A 3 (*Group*: NoREM, REM, WAKE) × 2 (*Session*: Session1, Session2) repeated-measures ANOVAs on the pre-testing sleepiness level, assessed using the Stanford Sleepiness Scale^19^, showed a significant reduction of sleepiness in Session 2 (5 PM) compare to Session 1 (9AM; F_(1,57)_ = 4.20, p = 0.045), which was mainly driven by the lower sleepiness level in the NoREM and REM (Fig. 2a). However this results was not statistically significant (Group × Session interaction: F_(2,57)_ = 1.83, p = 0.169). The same analysis of the fatigue levels, assessed used the Samn-Perelli scale^20^, showed a significant Session effect (F_(1,57)_ = 9.44, p = 0.003) and a significant Group × Session interaction (F_(2,57)_ = 6.12, p = 0.004), with NoREM and REM groups showing a post-nap reduction in the perceived fatigue level compared to the WAKE group (p = 0.008 and p = 0.074 respectively; Fig. 2b).

### Sacrificial moral dilemmas

Means and standard deviation for all the parameters of the sacrificial moral dilemmas are reported in Table 2.

**Table 2:** Means ± standard deviations for all the parameters of the sacrificial moral dilemmas in the three groups.

	Session	WAKE		NoREM		REM	
		Trolley Type	Footbridge Type	Trolley Type	Footbridge-Type	Trolley Type	Footbridge Type
Decision Choice	1	0.56 ± 0.26	0.12 ± 0.27	0.60 ± 0.30	0.06 ± 0.12	0.60 ± 0.27	0.14 ± 0.25
	2	0.53 ± 0.32	0.09 ± 0.23	0.57 ± 0.31	0.08 ± 0.18	0.68 ± 0.26	0.11 ± 0.19
Response Time (LN ms)	1	9.52 ± 0.53	9.15 ± 0.34	9.58 ± 0.41	9.31 ± 0.40	9.51 ± 0.33	9.13 ± 0.27
	2	9.46 ± 0.50	9.12 ± 0.35	9.40 ± 0.35	9.08 ± 0.40	9.16 ± 0.27	8.90 ± 0.30
Arousal	1	5.45 ± 1.50	5.56 ± 1.66	6.48 ± 1.51	6.30 ± 1.41	5.59 ± 1.65	5.26 ± 1.76
	2	5.80 ± 1.71	5.49 ± 1.70	6.36 ± 1.45	6.04 ± 1.56	5.04 ± 1.91	5.06 ± 1.99
Valence	1	2.38 ± 0.89	2.37 ± 1.02	2.07 ± 0.87	1.94 ± 0.73	2.73 ± 0.99	2.74 ± 1.19
	2	2.01 ± 1.08	2.31 ± 1.26	1.87 ± 0.67	2.00 ± 0.86	2.66 ± 0.95	2.62 ± 1.01
Moral Judgment	1	2.73 ± 1.35	2.02 ± 1.51	2.79 ± 1.55	2.12 ± 1.73	3.67 ± 1.67	2.80 ± 2.14
	2	2.42 ± 1.46	1.92 ± 1.64	2.57 ± 1.44	1.90 ± 1.91	3.02 ± 1.51	3.50 ± 1.70

LN: natural logarithm.

#### Decision choices

**Figure 3:**
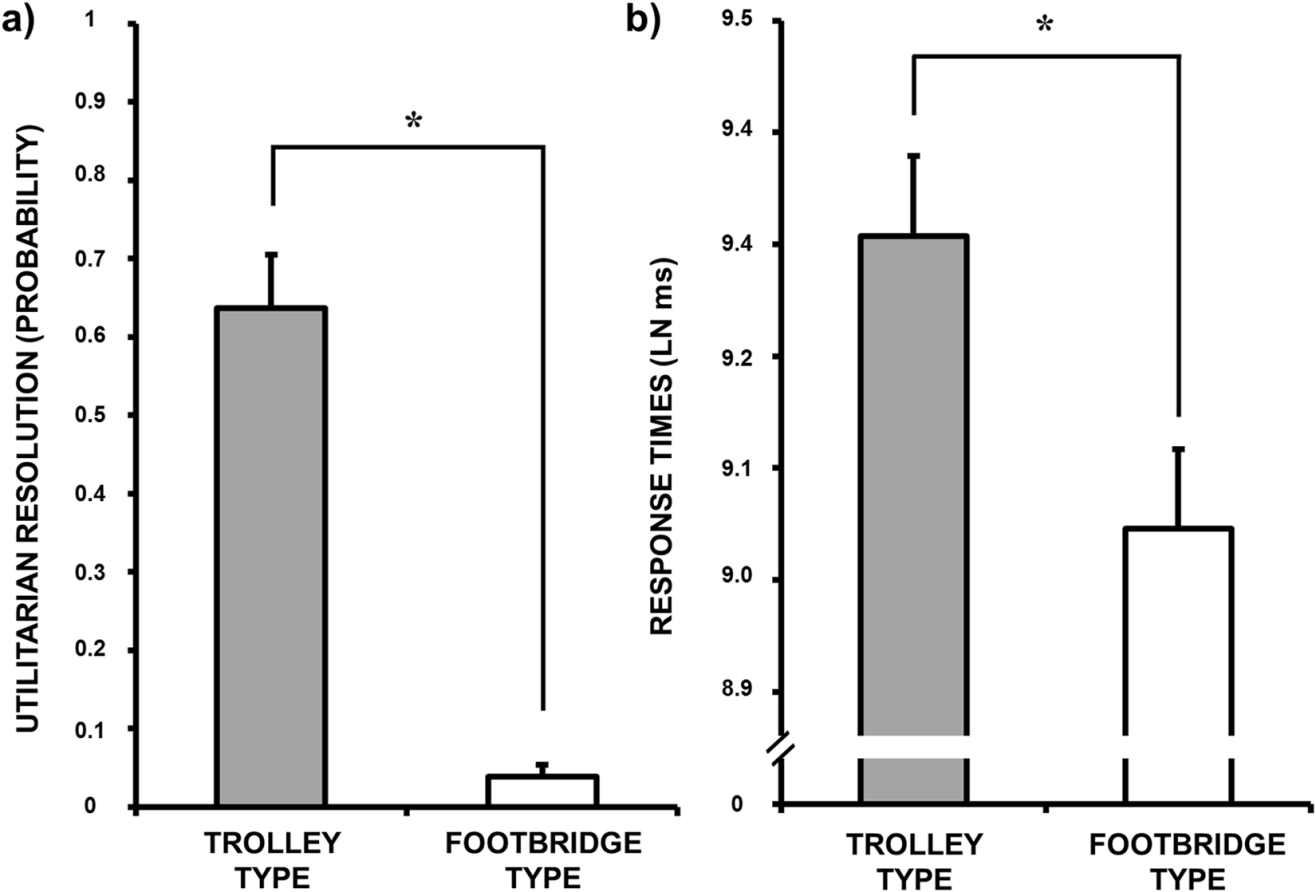
(**a**) Utilitarian resolution probability as a function of problem type (Trolley-type and Footbridge-type) in sacrificial moral dilemmas. (**b**) Response times as a function of problem type (Trolley-type and Footbridge-type) in sacrificial moral dilemmas. LN: natural logarithm. Error bars represent standard error. *p < 0.001.

Mixed-effect models, with *Participant* and *Moral Problems* as crossed random effects and *Problem Type* (Trolley-type/Footbridge-type dilemmas), *Session* (Session1/Session2), *Group* (REM/NoREM/WAKE), and their interactions as fixed effects, showed a significant *Problem Type* main effect (*χ* ^2^(1) = 40.49, *p* < 0.001), indicating that the probability of choosing the utilitarian resolution (i.e., sacrificing one individual to save more people) was significantly higher for Trolley than for Footbridge-type dilemmas (Fig. 3a). According to the approximate Bayes Factor, the posterior probability of choices being modulated by *Problem Type* was Pr(*H*_1_|*D*) ≈ 99% (BF_10_ > 150). The main effects of *Session* and *Group* and the interactions were not significant, with all the posterior probabilities being lower than Pr(*H*_1_|*D*) = 3% (all BF_10_s < 0.03), providing strong evidence against the alternative hypotheses.

#### Response times

Response times were log-transformed (natural logarithm) before the analyses. A significant *Problem Type* main effect was found (*χ* ^2^(1) = 23.91, *p* < 0.001; Pr(*H*_1_|*D*) ≈ 99%, BF_10_ > 150), with faster responses for the Footbridge- than for the Trolley-type dilemmas. The analysis also revealed a *Session* main effect (*χ* ^2^(1) = 54.13, *p* < 0.001; Pr(*H*_1_|*D*) ≈ 99%, BF_10_ > 150, Fig. 3b), with overall faster responses in Session 2 as compared to Session 1. The main effect of *Group* and the interactions were not significant, with all the posterior probabilities being lower than Pr(*H*_1_|*D*) = 3% (all BF_10_s < 0.03), indicating strong evidence against the alternative hypotheses.

#### Moral judgments

**Figure 4:**
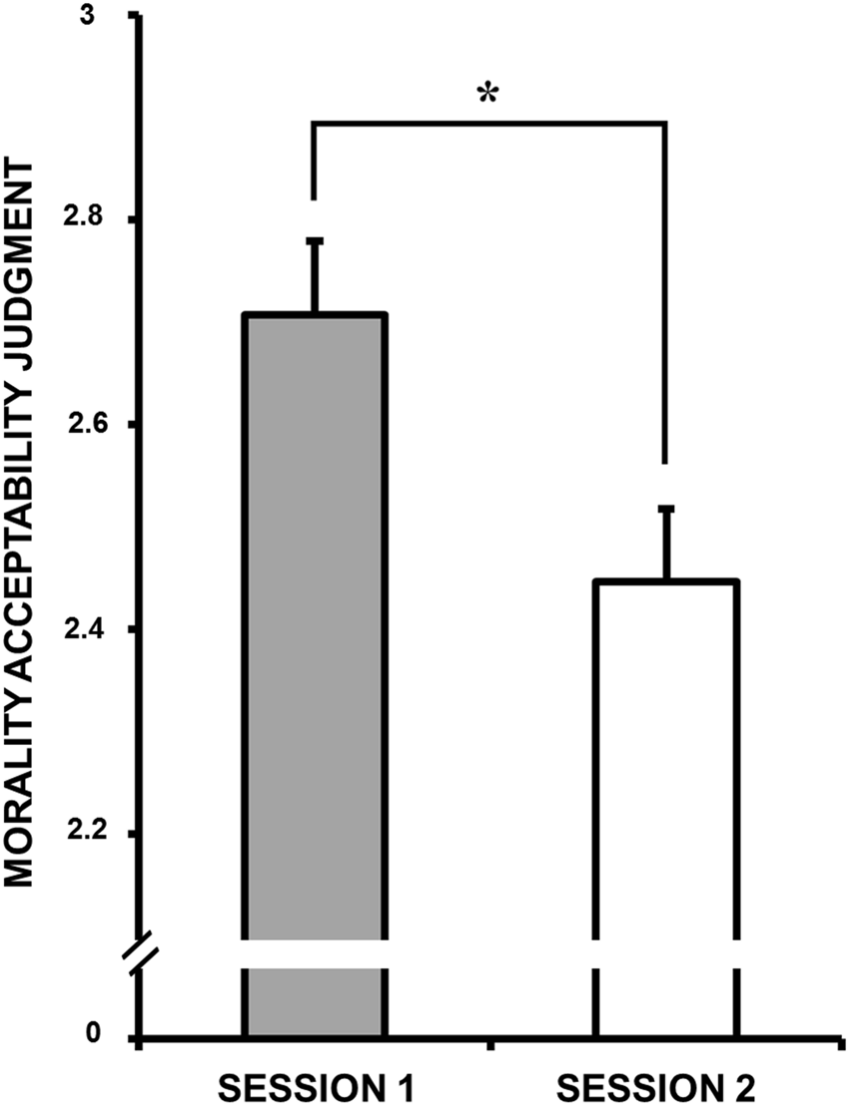
Moral judgment for sacrificial dilemmas as a function of Sessions. Error bars represent standard error. *p < 0.001.

A significant *Problem Type* main effect was obtained (*χ* ^2^(1) = 29.50, *p* < 0.001; Pr(*H*_1_|*D*) ≈ 99%; BF_10_ > 150), with utilitarian resolutions being rated as more morally acceptable for Trolley- than for Footbridge-type dilemmas. The *Session* main effect was also significant (*χ* ^2^(1) = 13.04, *p* < 0.001; Pr(*H*_1_|*D*) ≈ 95%, BF_10_ > 17.97; Fig. 4), with utilitarian resolutions being rated overall as less morally acceptable in Session 2. The other main effects and the interactions were not significant, with all the posterior probabilities being lower than Pr(*H*_1_|*D*) ≈ 5% (allBF_10_s < 0.06).

#### Affective ratings

No significant effects emerged for Valence ratings, with all the posterior probabilities being lower than Pr(*H*_1_|*D*) ≈ 3% (all BF_10_s < 0.15). As concerns Arousal ratings, the *Group* × *Session* interaction was significant (*χ* ^2^(1) = 40.49, p < 0.001), with REM group nominally decreasing their arousal ratings in the second session, but the approximate Bayes factor indicated strong evidence against the alternative hypotheses (Pr(*H*_1_|*D*) ≈ 3%, BF_10_ ≈ 0.04). No other effects were significant, with all the posterior probabilities being lower than Pr(*H*_1_|*D*) ≈ 11% (all BF_10_s < 0.12).

### Everyday moral conflict situations

Means and standard deviation for all the parameters of the everyday moral conflict situations are reported in Table 3.

**Table 3:** Means ± standard deviations for all the parameters of the everyday moral conflict situations in the three groups.

	Session	WAKE		NoREM		REM	
		Harmful	Harmless	Harmful	Harmless	Harmful	Harmless
Decision Choice	1	0.34 ± 0.28	0.40 ± 0.27	0.46 ± 0.25	0.36 ± 0.20	0.43 ± 0.29	0.25 ± 0.20
	2	0.44 ± 0.30	0.54 ± 0.23	0.57 ± 0.32	0.53 ± 0.22	0.37 ± 0.22	0.41 ± 0.26
Response Time (LN ms)	1	9.34 ± 0.44	9.26 ± 0.33	9.20 ± 0.31	9.13 ± 0.25	9.22 ± 0.36	9.21 ± 0.36
	2	9.24 ± 0.39	9.13 ± 0.36	9.35 ± 0.32	9.19 ± 0.31	9.07 ± 0.30	8.98 ± 0.26
Arousal	1	3.85 ± 1.60	3.39 ± 1.66	3.82 ± 1.20	2.88 ± 1.09	3.65 ± 1.77	3.50 ± 1.74
	2	3.88 ± 1.25	3.38 ± 1.03	3.78 ± 1.45	3.35 ± 1.48	3.87 ± 1.55	3.19 ± 1.69
Valence	1	4.49 ± 0.91	5.10 ± 0.90	4.62 ± 0.81	5.63 ± 1.21	4.91 ± 1.20	5.22 ± 0.97
	2	4.35 ± 0.92	5.00 ± 0.98	4.68 ± 0.95	5.62 ± 0.66	4.49 ± 1.17	5.35 ± 0.85
Moral Judgment	1	3.23 ± 1.44	4.19 ± 1.51	3.57 ± 1.30	5.24 ± 1.04	3.71 ± 1.45	5.63 ± 1.05
	2	3.14 ± 1.59	4.46 ± 1.50	3.41 ± 1.39	5.63 ± 1.21	3.50 ± 1.56	5.28 ± 1.06

LN: natural logarithm.

#### Decision choices

A significant *Session* main effect was obtained (*χ* ^2^(1) = 14.90, *p* < 0.001), indicating that the probability of choosing a moral violation was higher in Session 2 as compared to Session 1, with Pr(*H*_1_|*D*) ≈ 98% (BF_10_ > 55). The interaction between *Problem Type* and *Session* was significant (*χ* ^2^(2) = 3.81, *p* = 0.05), showing an increase in harmless moral violations in Session 2 as compared to Session 1. However, the posterior probability of decision choices being modulated by this interaction was low (Pr(*H*_1_|*D*) ≈ 18%; BF_10_ ≈ 0.22), suggesting that this result may be a spurious effect. The main effects of *Problem Type* and *Group*, and the other interactions were not significant, with all the posterior probabilities being lower than Pr(*H*_1_|*D*) ≈ 3% (all BF_10_s < 0.03), indicating strong evidence against the alternative hypotheses.

#### Response times

A significant *Session* main effect was found for response times (*χ* ^2^(1) = 8.32, *p* = 0.004; Pr(*H*_1_|*D*) ≈ 67%; BF_10_ = 2.08), with faster responses in Session 2 as compared to Session 1. The other main effects and interactions were not significant, with all the posterior probabilities being lower than Pr(*H*_1_|*D*) ≈ 3% (all BF_10_s < 0.03).

#### Moral Judgments

A significant *Problem Type* main effect was found (*χ* ^2^(1) = 24.18, *p* < 0.001; Pr(*H*_1_|*D*) ≈ 99%, BF_10_ > 150), with harmless moral violations being rated as more morally acceptable than harmful moral violations. A significant *Group* × *Problem Type* interaction was also observed (*χ* ^2^(2) = 7.86, p = 0.02; Fig. 4), which was driven by a nominal reduction of the moral acceptability in the harmless situations shown by the WAKE group, but the approximate Bayes factor indicated strong evidence against the alternative hypotheses (Pr(*H*_1_|*D*) ≈ 5%; BF_10_ ≈ 0.05). The *Session* and *Group* main effects and the other interactions were not significant, with all the posterior probabilities being lower than Pr(*H*_1_|*D*) ≈ 9% (all BF_10_s < 0.1).

#### Affective ratings

The analysis revealed a significant *Problem Type* main effect for Valence ratings (*χ* ^2^(1) = 9.47, p = 0.002; Pr(*H*_1_|*D*) ≈ 79%, BF_10_ ≈ 3.07), with participants reporting higher unpleasantness when deciding on harmful as compared to harmless moral situations. We did not observe any *Group* (*χ* ^2^(1) = 1.51, p = 0.047; Pr(*H*_1_|*D*) ≈ 2%, BF_10_ ≈ 0.002) or *Session* (*χ* ^2^(1) = 0.48, p = 0.049; Pr(*H*_1_|*D*) ≈ 4%, BF_10_ ≈ 0.04) effects.

A significant *Problem Type* main effect emerged also for Arousal ratings (*χ* ^2^(1) = 4.86, p = 0.03), with participants reporting higher arousal when deciding on harmful as compared to harmless moral situations. However, the approximate Bayes factor did not completely support the alternative hypotheses (Pr(*H*_1_|*D*) ≈ 27% BF_10_ ≈ 0.37). No *Group* (*χ* ^2^(1) = 2.54, p = 0.28, Pr(*H*_1_|*D*) ≈ 0.4%, BF_10_ ≈ 0.004) or *Session* (*χ* ^2^(1) = 0.74, p = 0.039; Pr(*H*_1_|*D*) ≈ 4%, BF_10_ ≈ 0.05) effect was present.

### Exploratory correlational analyses

**Figure 5:**
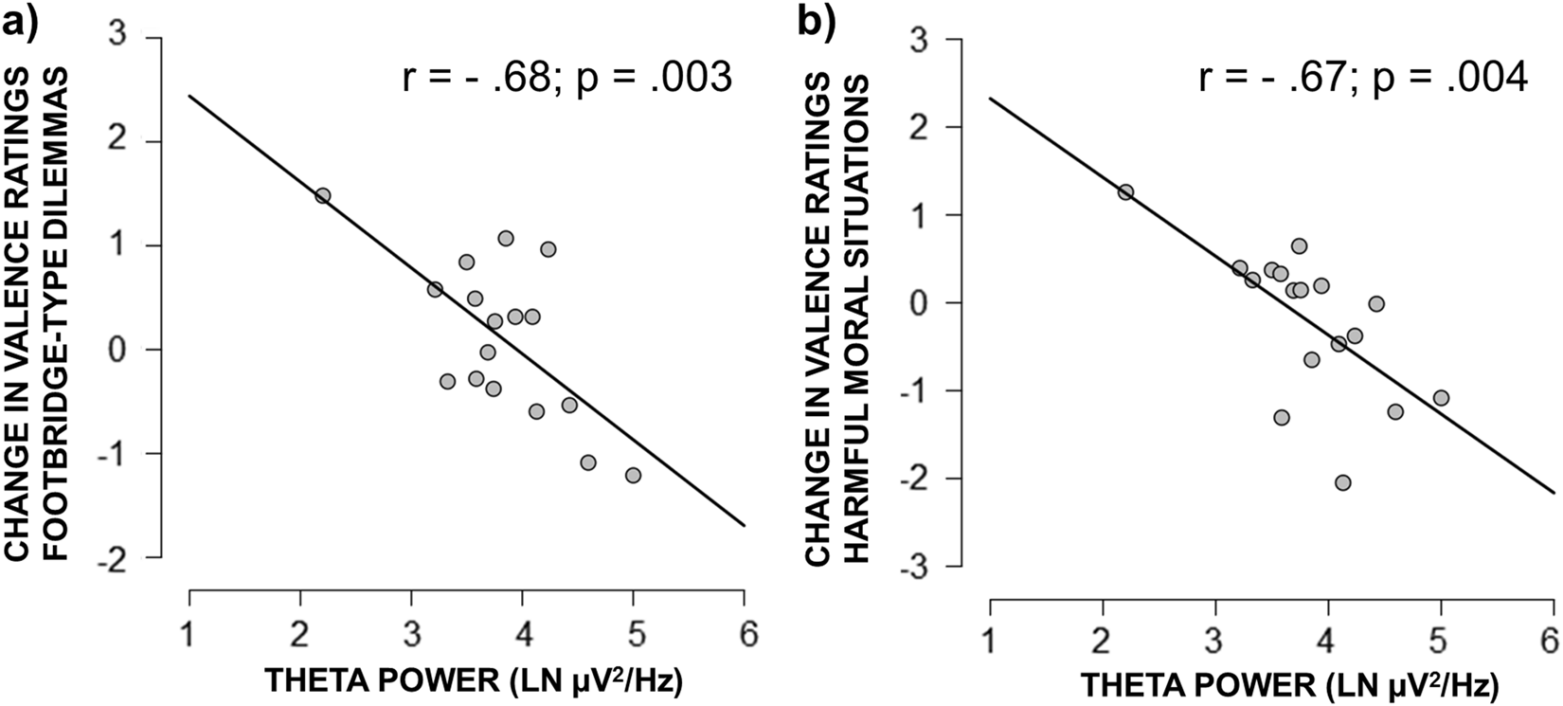
(**a**) Changes in Valence scores for Footbridge-type dilemmas as a function of theta power during REM sleep. (**b**) Changes in Valence scores for harmful moral violations as a function of theta power during REM sleep. LN: natural logarithm.

In the REM group, we observed a negative association between theta power (4.0–8.0 Hz) during REM sleep and changes in Valence scores between Session 1 and Session 2 for both Footbridge-type dilemmas (*r* = −0.66, *p* = 0.004; Fig. 5a) and harmful moral situations (*r* = −0.58, *p* = 0.015; Fig. 5b). This indicates that participants with higher theta power during REM sleep experienced increased unpleasantness when deciding on Footbridge-type dilemmas and harmful moral situations in Session 2 as compared to Session 1. No significant associations were observed between other task-related variables and sleep indices.

## Discussion

In the current study, we aimed to test the effect of a daytime nap on moral decision-making. Indeed, according to the dual-process theory of moral judgment^4, 5^, moral decision-making depends on the result of a competition between cognitive and emotional processing. A consequence of this theory is that reducing the emotional reaction to a moral situation should increase the probability of a utilitarian resolution of that situation. A physiological emotional regulation process seems to occur during sleep. Indeed, it has been suggested that while NREM sleep benefits the consolidation of declarative aspects of experiences, the subsequent REM sleep facilitates the processing of the emotional information, reducing the affective tone of these experiences^12, 13^.

On these grounds, we expected that a daytime nap characterized by the presence of REM sleep would impact moral decision-making, reducing the negative emotional reaction elicited by a moral dilemma and increasing the cognitive resources and, therefore, increasing the number of utilitarian resolutions of the moral dilemmas. However, our results did not support this hypothesis. Notwithstanding the fatigue accumulation throughout a working day (9 AM–5 PM) in the WAKE group, and although we observed that (1) participants judged the utilitarian choice as less morally acceptable in the afternoon and (2) theta activity during REM sleep was negatively correlated with the change in pleasantness when deciding how to resolve a dilemma, participants resolved the moral dilemmas in a consistent fashion at the morning and 8 hours later, independently of having slept in the mid-afternoon. In other words, we observed that neither the increase of fatigue (in the WAKE group), nor the change in the emotional evaluation of moral dilemmas (in the REM group), and nor the change in moral judgments (in all groups) were strong enough to impact the moral decision-making. It is worth mentioning that these negative findings were supported by Bayesian statistics, which constantly sustained the null hypothesis (i.e., no differences) against the H1. In case of null results, traditional null hypothesis testing does not allow us to state if the data support H0 or not. However, Bayes Factors do not suffer from this limitation: in fact, they permit estimation of the relative likelihood of the null and alternative hypotheses^21^. Thus, they provide valuable information in the context of null results, since they allow discernment of results that are really inconclusive –not clearly in favor of either hypothesis– from results that do support H0.

Here we showed that, consistent with the literature^22^, overall participants made less utilitarian choices for the Footbridge-type (more emotionally-driven) relative to the Trolley-type dilemmas (more cognitively-driven). Moreover, we observed a slower response time for the Trolley-type dilemma, indicating a more conflicting, and less automatic, decision processing for these dilemma, which resulted in more utilitarian responses.

Interestingly, participants rated the sacrificial dilemmas less moral in the afternoon than in the morning session, but this change in moral judgment did not modify the action choice that participants made. This dissociation between action choice and moral judgement, seems to be the results of different mechanism underlying two behaviors: the action choice depends on the outcome of a conflict between emotional and cognitive processing, with a major role played by the vmPFC, whereas moral judgments is mainly affected by cognitive aspect such as common norms and beliefs see refs 8, 23 and mainly relies on the activity of the DLPFC^24^. Therefore, it is possible that the passage of time, whether including a period of sleep or not, may have facilitated the elaboration of these dilemmas, allowing the participants to revise their judgments based on their social norms and beliefs. It is also possible that these outcomes may be the result of a learning effect. Indeed, although each moral dilemma is unique, the dilemmas within the same “Type” follow the same structure: e.g., in the Trolley-type dilemmas participants decide whether to indirectly kill someone to save a larger number of people. When participants are exposed to several dilemmas with the same structure, it is likely that they build a schema of the situations. Therefore, it is likely that learning processes are involved while making a moral decision in similarly structured dilemmas. That is, participants are likely not making a decision only based on the actual dilemma, but they may use previous decisions as a reference point in order to decide what is right or wrong, thereby influencing the decision at hand.

Similarly, the passage of time or a learning effect may have induced the changes we observed in the everyday-type moral violations. Indeed, participants endorsed more moral violations in the second session. It is possible that in the second session participants re-framed their decision based on the more dramatic decision that they had to make for the sacrificial dilemmas. This effect is particularly interesting, considering that everyday moral decisions do not seem to be susceptible to short-term modifications due to, for example, acute stress induction^25^. Future studies are needed to disentangle the relationship between passage of time and learning effects.

Our general idea was that daytime sleep, in particular REM sleep, would affect the emotional processing of these moral decisions since sleep is purported to reorganize schemas of emotional experiences^26^ and not of only single emotional engrams. An example of this reorganization has been observed in studies using fear-extinction protocols showing that sleep facilitates the generalization of extinction of conditioned fear to stimuli *similar* to the conditioned ones (for a review see ref. 14). In a similar way, we expected that sleep would generalize the affective regulation of highly emotionally experiences, such as moral dilemmas, via a reorganization of the emotional experience schemas. Specifically, one of our hypotheses was increased utilitarian choices after a nap containing REM sleep. Indeed, during REM, the brain networks underlying emotional processing and regulation (i.e., the amygdala, anterior cingulate cortex, and vmPFC) are activated to a greater extent than in wakefulness^14^, whereas the neural networks underlying executive functions and cognitive control (i.e., the DLPFC, posterior cingulate cortex, and inferior parietal cortex) are markedly deactivated. This brain activity may serve the reactivation and reprocessing of emotional information in amygdala-hippocampal-vmPFC networks, possibly mediated by theta activity^12–14, 17, 27^. According to the *Sleep to Remember, Sleep to Forget* hypothesis^12, 17^, this brain pattern is purported to reduce affective tone of emotional experiences, possibly through the enhancement of the functional connectivity of the vmPFC-amygdala circuit via theta oscillations which, by strengthening the vmPFC top-down control on the amygdala, may decrease brain reactivity to the subsequent emotional experiences of the waking state^13^.

However, here we observed the opposite effect, i.e., the more theta activity during REM, the higher increase unpleasantness when making a moral decision in both Footbridge-type dilemmas and harmful moral situations. This result might be more consistent with the “REM activation-attenuation” hypothesis proposed by Werner and colleagues^28, 29^, which suggested that REM sleep may impact emotional processing in a bi-phasic fashion. In detail, they suggest that REM sleep initially interferes with the habituation process that occurs during NREM sleep (see refs 30 and 31) and therefore strengthens the emotional salience of these memories. During the subsequent nights, when the declarative content of the initial information is already integrated with pre-existing networks, REM sleep may eventually lead to an attenuated emotional response.

Also, Hutchison & Rathore^27^ suggested that REM may be complementary to NREM sleep in integrating recently encoded information in the neocortex via theta activity. However, in their view, while on the one hand the typical neurochemical milieu of REM sleep (i.e., high level of acetylcholine coupled with low level of noradrenaline) may facilitate the cortical integration of pre-existing memories (e.g., dogs love to be petted) with recent information (e.g., my friend’s dog growls when you try to pet it), on the other hand the emotional network activation may optimize (either preserving or changing) the emotionally-driven behavior related to this information (e.g., don’t touch my friend’s dog!).

Finally, it has been reported that decision-making is affected by daylong fatigue accumulation, which impairs the activity in pre-frontal areas^32^. However, here we observed no effect of fatigue build-up throughout a “typical” working day (9 AM–5 PM) on moral decision-making. This result is consistent with previous studies testing the impact of sleep deprivation on moral judgment^33–35^, suggesting, together with the absence of any sleep effect, that moral decision-making is not sensitive to short-term changes. Future studies should test whether individual moral decision-making style is stable over time.

Some limitations of the present study are worth acknowledging. First, we used a classic nap paradigm with the aim of obtaining sleep with and without REM in an ecological way^16, 36^. This method, although successful, limits the amount of REM sleep that a participant can obtain in a nap. Therefore, it is possible that a stronger REM effect may arise after a relatively higher amount of sleep spent in this stage. Also, daytime naps are characterized by different underlying physiological activity following a circadian rhythm, such as cortisol level and body temperature, compared to nocturnal sleep, which may result in differential emotional processing (see ref. 37). In this view, future research should assess the change in moral judgment and decisions after several days, during which an individual would experience several REM sleep cycles (including nocturnal REM). Second, we used a limited number of dilemmas per session (i.e., 20 dilemmas), which may have reduced our statistical power. However, it should be noted that the number of dilemmas used in our study is higher than the average number of dilemmas used in behavioral studies in the existing literature on moral dilemmas. Indeed, several of the key studies in this field used only one dilemma per category^3, 11, 38, 39^. Also, using a high number of dilemmas has some drawbacks, because participants might get used to the stimuli, given that the dilemma structure is the same, and start to automatize judgment and decisions. Nevertheless, the use of both linear-mixed modeling combined with Bayesian statistics, which constantly sustained the null hypothesis (i.e., no differences) against the alternative hypothesis, allowed us to compensate for the reduced number of trials.

In conclusion, we observed that within a day, when individuals are re-exposed to moral dilemmas, their judgment became more severe. Moreover, we showed that after a daytime nap participants with higher theta power during REM sleep experienced an increased unpleasantness during Footbridge-type dilemmas and harmful moral situation. Nevertheless, moral decision-making did not change across a day, and it was not modulated by sleep or specific sleep-stage physiology. These results suggest that although both time and REM sleep may affect the evaluation of a moral situation, these factors did not ultimately impact the individual moral choices.

## Methods

### Participants

Sixty healthy university students (33 female) between the ages of 19 and 29 years (22.73 ± 2.25 years) participated in the study. All participants were enrolled by way of advertisements posted at the University of Padova, were right-handed, had no history of psychiatric, neurological, or sleep disorders, and had normal or corrected-to-normal vision. They were paid 13€ for their participation. The study was approved by the Ethics Committee of the Departments of Psychology, University of Padova, and was in line with the Declaration of Helsinki. All participants gave written consent before participation.

### Stimulus Material and Experimental Task

We employed two sets of 20 hypothetical moral problems, each including 12 *sacrificial* moral dilemmas, in which the agent must choose whether or not to sacrifice one individual to save more people, and 8 *everyday* moral conflict situations, in which the agent must choose whether or not to violate a moral obligation toward others in order to pursue a personal advantage. The 12 sacrificial dilemmas were selected from the standardized set from Lotto *et al*.^40^; Fig. 1a and included 6 *Footbridge-type* dilemmas, describing sacrificing one individual as an intended means to save others, and 6 *Trolley-type* dilemmas, describing sacrificing one individual as a foreseen but unintended consequence of saving others. Detailed information on the criteria used to develop this set of stimuli can be found in Sarlo *et al*.^6^ and in Lotto *et al*.^40^. The sacrificial moral dilemmas of the two sets of stimuli were comparable for numerical consequences (i.e., the number of people to save or let die) and normative arousal and valence ratings. The 8 everyday situations cf.^23^ were developed to be structured similar to the sacrificial dilemmas, but involved no deaths and described a personal advantage either involving harm to another’s good (4 *harmful situations*, e.g., stealing) or not posing negative consequences for others (4 *harmless situations*, e.g., lying). The everyday situations of the two sets of stimuli were comparable in terms of normative arousal and valence ratings.

Each moral problem was presented as text through a series of two screens (see Fig. 1b). The first one described the scenario and the second one described a hypothetical action that could be performed (i.e., a utilitarian resolution for sacrificial dilemmas, and a moral violation for everyday situations). Participants had to choose whether or not they would perform the proposed action by pressing one of two buttons as “Yes” or “No”. After their response, participants were required to rate how they felt while they were deciding using a computerized version of the Self-Assessment Manikin^41^, displaying the 9-point scales of valence (unpleasantness/ pleasantness) and arousal (calm/ activation), with higher scores indicating higher pleasantness and higher emotional arousal. Then, participants rated to what extent the proposed action was morally acceptable on a scale from 0 (not at all) to 7 (completely), independently of whether they decided to perform it. The task lasted about 20 minutes.

The two sets of moral problems were presented counterbalanced across participants in two separate sessions (held at 9 AM and at 5 PM), in randocrime software 2.0 (Psychology Software Tools, Pittsburgh, PA).

### Procedure

During the recruitment phase, participants were told that they had to perform two experimental sessions, a moral decision task in the morning, and an attentional task in the afternoon. This was done to avoid participants to voluntarily rehearse the moral dilemmas and their resolutions. They were also told whether they had to take a midday nap (NAP group) or to leave the lab and continue with their normal daytime activities between the two experimental sessions (WAKE group).

Participants arrived in the lab at 9 AM (Fig. 1a), where they were given information about the experiment, their written informed consent was obtained, and information about their sleepiness and fatigue level were obtained using the Stanford Sleepiness Scale^19^ and the Samn-Perelli scale^20^, two single-item 7-point scales. Shortly afterwards they were presented with the first set of moral problems (Session 1).

Afterwards, all participants were allowed to leave the lab and continue with their normal daytime activities, but participants in the NAP group returned to the lab at 12 PM and took either a 90-min or a 120-min polysomnographic-recorded nap.

At 5 PM all participants were presented the second set of moral problems (Session 2), after which they were debriefed and allowed to leave the lab.

### Polysomnography

Polysomnographic (PSG) recordings were conducted according to the American Academy of Sleep Medicine (AASM) guidelines^42^ by using Compumedics Siesta 802 acquisition system (Compumedics, Abbotsford, Australia) with 6 electroencephalographic (EEG) channels (F3-A2, F4-A1, C3-A2, C4-A1, O1-A2, O2-A1), electrooculogram and bipolar submental electromyogram. EEG signals were amplified, band-pass filtered (0.3–35 Hz) and digitalized at 512 Hz. Sleep stages (wake, N1, N2, N3, REM) were visually scored using 30-sec epochs according to the AASM rules^42^.

Based on prior studies about the role of sleep spindles frontal slow wave activity (SWA; 0.5–4.0 Hz) and theta activity (4.0–8.0 Hz) in both declarative and emotional memory processing^17^, we computed the power density (μV^2^/Hz) of the frontal SWA and of the theta activity during artifact-free 30-sec epochs of N3 and REM sleep, respectively. Similarly, since literature has shown that sleep spindles activity may play a role in processing cognitive and emotional information^43, 44^, we computed the number of spindles and spindle density (i.e., number of spindles relative to the minutes spent in a specific sleep stage) for N2 sleep using an automated spindle detection method see ref. 45. For further detail on the spindle detection and spectral method see Cellini *et al*.^46^.

### Statistical analysis

Two separates 3 (*Group*: NoREM, REM, WAKE) × 2 (*Session*: Session1, Session2) repeated-measures ANOVAs were performed to assess changes in fatigue and sleepiness value across the experimental day.

We performed separate statistical analyses on sacrificial moral dilemmas and everyday moral conflict situations, as these two types of moral problems are not directly comparable. The data were analyzed using mixed-effect models, which eliminate the need of averaging scores across trials and permit the inclusion of each single observation in the analyses, thus increasing statistical power. Moreover, mixed models allow us to take into account not only the influence of fixed effects manipulated by the experimenter (e.g., problem type and group) but also the influence of factors whose levels are randomly extracted from a population (i.e., participants, individual moral problems), thus yielding more generalizable results^47^.

For each dependent variable (decision choices, response times, moral judgment, and ratings of valence and arousal) we built a separate set of models, using *Participant* and *Moral Problems* as crossed random effects and *Problem Type* (Trolley-type/Footbridge-type dilemmas, or harmless/harmful situations), *Session* (Session1/Session2), *Group* (REM/NoREM/WAKE), and their interactions as fixed effects. To compare models, we used the log-likelihood ratio test.

Additionally, in order to provide further information on the probability of the effects given the data [Pr(*H*_1_|*D*)], we calculated the approximate Bayes Factor (BF_10_) through the Bayesian Information Criterion (BIC), following the procedure described in Wagenmakers^48^. This is especially useful in case of null results because it allows estimation of the probability of the null hypothesis being true given the data. A BF_10_ score higher than 20 indicates strong evidence in support of the alternative hypothesis, whereas a score lower than 0.03 indicates strong evidence in support of the null hypothesis. BF_10_ scores between 0.03 and 30 indicate no to moderate evidence in support of the alternative hypothesis (BF_10_ ≥ 1) or the null hypothesis (BF_10_ ≤ 1)^21, 48^.

Pearson’s correlations were used to examine the relationship between changes in behavioral responses or subjective emotional reactivity and specific sleep features. Changes in behavioral responses and subjective emotional reactivity were computed using the residuals from a linear regression of Session 2 values on Session 1 values. This method allows the removal of variance in Session 2 values that could be accounted for by Session 1, leaving the residuals as a pure measure of change in variable scores, uninfluenced by differences in baseline values see^49, 50^.

All statistical analyses on behavioral data were performed in R^51^, using the libraries *stats* ^51^, *lme4* ^52^, and *effects* ^53^.
